# Supplementary material for: Association between Preoperative Retrograde Hepatic Vein Flow and Acute Kidney Injury after Cardiac Surgery
Source: Diagnostics (Basel). 2022 Mar 12;12(3):699. doi: 10.3390/diagnostics12030699 (PMC8946915; doi:10.3390/diagnostics12030699)
Supplement: Supplementary file 1 [file diagnostics-12-00699-s001.zip › diagnostics-1586288-supplementary.pdf]

Supplement – Table S1: Acute kidney injury and postoperative parameters.

	All patients	%ΔCr B	95% CI		P value	AKI	non-AKI	p value
**Mechanical ventilation (hours)**	46.1 (9.5)	0.026	-0.002	0.054	0.066	47.1 (9.2)	46.9 (10.1)	0.076
**Need for vasopressor/inotrope**	29 (32%)	0.282	-0.271	0.836	0.314	11 (64%)	18 (23%)	0.234
**VIS**	4.2 (8.2)	0.008	0.000	0.017	0.057	4.9 (8.1)	3.6 (8.2)	0.122
**Need for dialysis**	3 (3.7%)	0.021	-0.012	0.022	0.065	2 (2.4%)	1 (1.3%)	0.09
**ICU days**	2.2 (0.9)	0.277	-0.223	0.766	0.344	2.3 (1.0)	2.2 (0.9)	0.123
**Hospital days**	12.8 (5.6)	0.123	-0.156	0.544	0.231	14 (6.5)	12 (8.7)	0.245
**Transfusion RBC (U)**	0 (0-2)	0.043	0.008	0.078	0.016	2.2 (3.1)	0.9 (1.9)	0.002
**CVP (max) (Hgmm)**	9.0 (2.5)	0.009	-0.005	0.023	0.197	11.0 (2.0)	8.0 (1.2)	0.322
**Fluid balance (ml/kg)**	34.8 (29.1)	0.0001	-0.005	0.023	0.068	50.1 (40.1)	19.6 (3.4)	0.098
**Furosemide (mg)**	25.0 (10.0)	0.004	0.003	0.006	0.0001	40.0 (10.0)	20.0 (10.0)	0.001

Acute kidney injury was determined by the KDIGO AKI criteria and by the as the percentage change of the highest postoperative serum creatinine from the baseline preoperative concentration (%ΔCr). Data are expressed as number and (%) or mean and (standard deviation).

VIS: Vasoactive-inotropic score

RBC: Red blood cell

CVP: Central venous pressure

FSD: Furosemide

Supplement – Table S2: Univariable linear regression calculations on the ratio of retrograde/antegrade wave VTI

	All patients	AKI	non-AKI	P value	B	95% CI lower	upper	P value
**Age (years)**	68.8 (11.2)	69.1 (7.4)	63.5 (13.9)	0.09	0.004	0.086	0.351	0.247
**Weight (kg)**	74.6 (8.1)	72.6 (7.1)	75.1 (9.1)	0.65	0.000	-0.009	0.008	0.936
**Diabetes**	20 (20%)	5 (25%)	15 (19%)	0.43	-0.110	-0.317	0.097	0.295
**Atrial fibrillation**	32 (33%)	9 (23%)	23 (77%)	0.32	0.090	-0.093	0.273	0.332
**PulmHT**	7 (8%)	1 (14%)	6 (86%)	0.07	-0.209	-0.664	0.245	0.363
**MELD**	7.6 (19.0)	7.50 (4.6)	7.61 (31.2)	0.5	-0.001	-0.004	0.003	0.724
**EF**	54.9 (10.6)	54.0 (11.6)	55.1 (12.6)	0.49	-0.001	-0.008	0.005	0.651
**TAPSE (mm)**	23.6 (8.5)	22.1 (8.1)	24.2 (8.8)	0.4	-0.008	-0.0023	0.007	0.282
**RASA (mm2)**	1911.1 (176.1)	1935.3 (200.1)	1835.1 (199.3)	0.06	-0.004	0.000	0.207	0.045
**RV (mm)**	30.9 (8.0)	29.7 (8.9)	32.7 (6.5)	0.14	0.008	-0.004	0.003	0.304
**Na (mmol/l)**	141.2 (3.1)	142.1 (3.9)	137.8 (3.4)	0.53	0.001	-0.004	0.007	0.629
**INR**	1.9 (10.5)	1.1 (0.3)	3.9 (22.2)	0.32	-0.001	-0.005	0.004	0.792
**Alb (g/l)**	41.4 (8.6)	38.8 (8.1)	43.7 (7.9)	0.83	-0.010	-0.022	0.002	0.096
**Creatinine (umol/l)**	87.8 (20.1)	109.8 (25.1)	78.4 (17.7)	0.001	-0.002	-0.005	0.002	0.353
**BUN (mmol/l)**	6.4 (2.1)	5.9 (1.8)	7.4 (3.2)	0.18	-0.001	-0.041	0.040	0.965
**GFR**	69.7 (15.4)	77.2 (15.6)	60.0 (16.6)	0.001	0.003	-0.002	0.008	0.196
**WBC (G/l)**	7.92 (1.2)	7.65 (1.1)	8.11 (1.2)	0.45	-0.001	-0.008	0.005	0.067

**HGB (g/l)**	137.1 (18.5)	135.3 (19.7)	138.4 (18.7)	0.85	-0.002	-0.006	0.000 2	0.372
**THR (G/l)**	217.5 (62.2)	218.6 (64.9)	211.4 (58.7)	0.41	0.000	-0.001	0.001	0.812
**EuroSCORE**	1.6 (0.9)	1.6 (1.0)	1.5 (0.7)	0.09	0.006	-0.0370	0.048	0.794
**Operation time (min)**	182.4 (39.1)	178.1 (41.1)	188.8 (39.1)	0.89	0.001	-0.001	0.003	0.243
**Aorta clamp time (min)**	47.8 (7.1)	40.8 (9.1)	48.1 (7.6)	0.73	0.000	-0.002	0.002	0.796

Afib: Atrial fibrillation

PulmHT: Pulmonary hypertension

HGB: Hemoglobin

THR: Thrombocyte

CRP: C-reactive protein

INR: Internationally normalized ratio

ASAT: Aspartate aminotransferase

ALAT: Alanine aminotransferase

Creat: Creatinine

BUN: Blood urea nitrogen

GFR: Glomerular filtration rate

SeBi: Serum bilirubin

MELD: Model for end-stage liver disease

RASA: Right atrium systolic area

RV: Right ventricle

Supplement – Table S3: Echocardiographic parameters

		**All patients**	**AKI**	**non-AKI**	**p**
**EF**	**preoperative**	54.9 (10.6)	54.0 (11.6)	55.8 (12.3)	0.49
	**postoperative**	57.3 (8.1)	55.6 (8.9)	58.4 (7.1)	0.77
**TAPSE (mm)**	**preoperative**	23.6 (8.0)	22.8 (8.8)	24.1 (8.9)	0.4
	**postoperative**	13.4 (8.1)	13.4 (7.6)	14.6 (8.6)	0.27
**LVEDD (mm)**	**preoperative**	50.3 (6.1)	50.5 (6.2)	49.6 (5.5)	0.73
	**postoperative**	46.0 (8.3)	44.6 (9.1)	46.3 (7.1)	0.97
**LVESD (mm)**	**preoperative**	35.8 (5.1)	36.1 (4.3)	34.3 (5.3)	0.38
	**postoperative**	34.8 (7.8)	36.1 (7.4)	33.0 (6.4)	0.92
**RV (mm)**	**preoperative**	30.8 (8.1)	29.2 (8.8)	32.7 (6.5)	0.14

	**postoperative**	33.2 (9.0)	32.3 (9.0)	33.7 (9.1)	0.96
**LA (mm)**	**preoperative**	43.1 (8.3)	41.8 (8.5)	43.4 (7.0)	0.44
	**postoperative**	43.3 (6.3)	43.7 (8.6)	43.2 (3.7)	0.31
**RA (mm)**	**preoperative**	40.5 (7.6)	38.0 (7.2)	41.9 (8.7)	0.66
	**postoperative**	43.2 (6.2)	44.1 (7.0)	43.2 (6.7)	0.54

EF: ejection fraction
TAPSE: triscuspidal anular plane systolic excursion
LVEDD: left ventricle end-diastolic diameter
LVESD: left ventricle end-sysstolic diameter
RV: right ventricle
LA: left atrium
RA: right atrium

Supplement – Table S4: Descriptive analysis of parameters, missing values

Pre and intraoperative variables	**All patients**	**n**	**Missing values**	**Postoperative**	**n**	**Missing values**
**Age (years)**	68.8 (11.2)	95	0	**Mechanical ventilation (hours)**	93	2
**Weight (kg)**	74.6 (8.1)	95	0	**Need for vasopressor/inotrope**	95	0
**Diabetes**	20 (20%)	95	0	**VIS**	95	0
**Atrial fibrillation**	32 (33%)	95	0	**Need for dialysis**	94	1
**PulmHT**	7 (8%)	95	0	**ICU days**	95	0
**MELD**	7.6 (19.0)	95	0	**Hospital days**	95	0
**EF**	54.9 (10.6)	94	1	**Transfusion RBC (U)**	93	2
**TAPSE (mm)**	23.6 (8.5)	94	0	**CVP (max) (Hgmm)**	95	0
**RASA (mm2)**	1911.1 (176.1)	94	1	**Fluid balance (ml/kg)**	95	0
**RV (mm)**	30.9 (8.0)	94	1	**Furosemide (mg)**	93	2
**Na (mmol/l)**	141.2 (3.1)	95	0			
**INR**	1.9 (10.5)	95	0			
**Alb (g/l)**	41.4 (8.6)	95	0			
**Creatinine (umol/l)**	87.8 (20.1)	95	0			
**BUN (mmol/l)**	6.4 (2.1)	95	0			
**GFR**	69.7 (15.4)	95	0			
**WBC (G/l)**	7.92 (1.2)	95	0			
**HGB (g/l)**	137.1 (18.5)	95	0			

**THR (G/l)**	217.5 (62.2)	95	0			
**EuroSCORE**	1.6 (0.9)	95	0			
**Operation time (min)**	182.4 (39.1)	95	0			
**Aorta clamp time (min)**	47.8 (7.1)	95	0			

EF: Ejection fraction

TAPSE: Triscuspidal anular plane systolic excursion

RASA: Right atrium systolic area

RV: Right ventricle

PulmHT: Pulmonary hypertension

HGB: Hemoglobin

THR: Thrombocyte

CRP: C-reactive protein

INR: Internationally normalized ratio

Alb: Albumine

BUN: Blood urea nitrogen

GFR: Glomerular filtration rate

VIS: Vasoactive-inotropic score

RBC: Red blood cell

CVP: Central venous pressure
